# MethylDetectR: a software for methylation-based health profiling

**DOI:** 10.12688/wellcomeopenres.16458.2

**Published:** 2021-04-13

**Authors:** Robert F. Hillary, Riccardo E. Marioni

**Affiliations:** 1 Centre for Genomic and Experimental Medicine, Institute of Genetics and Molecular Medicine, University of Edinburgh, Edinburgh, Midlothian, EH4 2XU, UK

**Keywords:** epigenetics, DNA methylation, epidemiology, translation, Shiny, prediction

## Abstract

DNA methylation is an important biological process that involves the reversible addition of chemical tags called methyl groups to DNA and affects whether genes are active or inactive. Individual methylation profiles are determined by both genetic and environmental influences. Inter-individual variation in DNA methylation profiles can be exploited to estimate or predict a wide variety of human characteristics and disease risk profiles. Indeed, a number of methylation-based predictors of human traits have been developed and linked to important health outcomes. However, there is an unmet need to communicate the applicability and limitations of state-of-the-art methylation-based predictors to the wider community. To address this need, we have created a secure, web-based interactive platform called ‘MethylDetectR’ which automates the calculation of estimated values or scores for a variety of human traits using blood methylation data. These traits include age, lifestyle traits and high-density lipoprotein cholesterol. Methylation-based predictors often return scores on arbitrary scales. To provide meaning to these scores, users can interactively view how estimated trait scores for a given individual compare against other individuals in the sample. Users can optionally upload binary phenotypes and investigate how estimated traits vary according to case vs. control status for these phenotypes. Users can also view how different methylation-based predictors correlate with one another, and with phenotypic values for corresponding traits in a large reference sample (n = 4,450; Generation Scotland). The ‘MethylDetectR’ platform allows for the fast and secure calculation of DNA methylation-derived estimates for several human traits. This platform also helps to show the correlations between methylation-based scores and corresponding traits at the level of a sample, report estimated health profiles at an individual level, demonstrate how scores relate to important binary outcomes of interest and highlight the current limitations of molecular health predictors.

## Introduction

DNA methylation (DNAm) is an epigenetic mechanism in which methyl groups are added to the genome sequence. Inter-individual variability in DNAm profiles results from differences in both underlying genetics and environmental influences ^1^. Factors such as diet, stress and smoking behaviours may influence the process of methylation. Typically, methyl groups are added to cytosine residues in the context of a cytosine-guanine dinucleotide (CpG site) ^2^. The addition of these chemical tags can alter whether, and to what extent, a gene is active. In contrast to genetics, these molecular modifications are dynamic, tissue-specific and reversible ^3^. Further, methylation at many CpG sites is tissue-specific though some show strong concordance across multiple tissues ^4^. In addition, CpG modifications induced through environmental factors, such as smoking, may be reversible or show persistent alterations ^5^.

Biological data may be harnessed to estimate or predict a variety of human characteristics and disease risk profiles. There is a growing body of evidence demonstrating the effective creation and application of DNAm-based predictors of human traits and health ^6– 19^. Additionally, methylation-based predictors of traits such as smoking status may provide more accurate measurements than self-reported information, thereby allowing for improved disease prediction and risk stratification ^20^. Blood DNAm data is often used as it is minimally-invasive to collect and it provides a good index of the overall health status of the body ^21^.

Increased training sample sizes and refinements in statistical and machine learning methodologies have improved the accuracy of DNAm-based predictors ^22, 23^. Furthermore, there has been an increase in the commercialisation and scalability of DNAm assays for direct-to-consumer use or for use in clinical, research or industrial settings ^24^. A major goal of using these predictors is to aid in prediction strategies and provide better clinical outcomes for individuals. Therefore, translational platforms for methylation-based health profiling are warranted in order to communicate the applications and limitations of DNAm-based predictors of human traits.

To address this need, we have created a web-based platform called ‘MethylDetectR’ that allows for an interactive demonstration of state-of-the-art DNAm-based predictors. A demo version of the app which does not require the upload of data is available at: https://shiny.igmm.ed.ac.uk/MethylDetectR_Demo/. The DNAm-based predictors in this platform include a highly accurate predictor of chronological age trained in > 13,000 individuals across 14 cohorts with a root mean squared error of 2.04 years in the original publication ^22^. We also include six DNAm-based predictors of lifestyle and biochemical traits: alcohol consumption per week, body fat percentage, body mass index, high-density lipoprotein (HDL) cholesterol, smoking status and waist-to-hip ratio ^25^. These predictors were generated in 5,087 individuals who are members of the Generation Scotland: Scottish Family Health Study (GS) which represents one of the largest DNAm resources in the world.

Briefly, the ‘MethylDetectR’ platform consists of two applications. The first application named ‘MethylDetectR – Calculate Your Scores’ allows users to securely upload Illumina 450k or EPIC DNAm array data and obtain blood-based methylation predicted scores (or values) for the aforementioned traits. No data are stored by the application. Furthermore, predicted scores are often returned on arbitrary scales. The use of this application is optional as users may instead use R scripts which we have made publicly available if they so wish or if their DNAm files are too large for upload to the online application (>3 GB) ( https://doi.org/10.5281/zenodo.4646300). However, users can access a file called ‘Truncate_to_these_CpGs.csv’ to subset the list of CpG sites in their DNAm files to those required by the ‘MethylDetectR – Calculate Your Scores’ application. This should reduce file size and upload time. The second and main application named ‘MethylDetectR’ allows users to compare DNAm-derived scores for any individual in their input dataset against other individuals in the input dataset. Percentile ranks for individuals in the input dataset may be downloaded. Users can also upload an optional file containing binary phenotypes whereby individuals are coded as ‘0’ for control status and ‘1’ for case status. This information allows users to view how distributions of the DNAm-derived traits vary by cases and controls. Users can also view how the various DNAm-based predictors correlate with one another in the input dataset and in a separate reference sample. This reference sample comprises 4,450 individuals who are members of the GS study. These individuals are unrelated to each other and distinct from those in the original training samples in which the predictors for age, HDL cholesterol and lifestyle traits were developed. They are also unrelated to those included in the original training sample. Furthermore, information is provided on how well the predicted scores correlate with phenotypic values for corresponding traits that are available in GS. Lastly, the user can subset the input sample by sex, age range or case vs. control status determined by the case-control variables uploaded by the user. Further, the user can subset the GS reference sample by age and sex. 

This platform communicates important information relating to the generation and applicability of DNAm-based predictors of human traits and health. The ‘MethylDetectR’ platform also represents a research tool for fast and automatic generation of DNAm-derived estimates for human traits. This platform can show that DNAm-based scores for traits may correlate well with measured values for a given trait at the level of the cohort. For example, a predictor for epigenetic age correlates strongly with true age with a root mean squared error of 2.04 years ^22^. However, this platform also helps to show that predictors may report inaccurate values at an individual level. For instance, although the age predictor correlates well with true age at the level of the cohort, an individual’s predicted age may differ from their true age by a number of years or decades. The optional incorporation of binary phenotype data allows users to view how well established or putative risk factors, as estimated by DNAm data, are stratified according to cases and controls for a given trait of interest. Together, the functionalities of ‘MethylDetectR’ begin to address the translational gap in the development and implementation of molecular-based health predictors by highlighting their performance and limitations in advance of their potential utility in diagnostic and stratification paradigms.

## Methods

### Implementation

***Data protection and privacy.*** No data are stored in ‘MethylDetectR’ and are deleted upon closing the applications. Applications are also timed out after three minutes of inactivity and are hosted on patched and secure servers within the Institute of Genetics and Cancer, University of Edinburgh. This research and translational tool complies with GDPR guidelines and has been designed to ensure the highest level of data security and privacy. The ‘MethylDetectR’ applications and information on their usage are also available at the following website: https://www.ed.ac.uk/centre-genomic-medicine/research-groups/marioni-group/methyldetectr. Information relating to participant consent is also available at this website. Given that no data are stored, this information pertains to general risk surrounding the upload of biological data to online software and the measures taken to mitigate the risk of motivated intruders gaining access to such data.

***The ‘MethylDetectR’ platform.*** The ‘MethylDetectR’ platform consists of two applications. The first application is called ‘MethylDetectR - Calculate Your Scores’. Users may upload DNAm data as an R object (.rds file) and obtain estimated values or scores for a variety of traits across individuals in their input dataset ( https://shiny.igmm.ed.ac.uk/Calculate_Your_Scores/). The upload limit is 3 gigabytes; however, files greater than 500 megabytes may take a considerable amount of time to upload. Users can make these upload files smaller by subsetting to CpG sites used in ‘MethylDetectR – Calculate Your Scores’. These CpG sites are available in the ‘Truncate_to_these_CpGs.csv’ file in Zenodo (Zenodo link). An optional ‘SexAgeInfo’ file may also be uploaded in order to include sex and age information in the output file. This should be a .csv file and have three columns: one column for the IDs of individuals in the methylation file (‘ID’ column), one column should list the sex of these individuals written as ‘Male’ or ‘Female’ or ‘NA’ (‘Sex’ column) and one column should report the actual or chronological age of individuals (‘Age’ column). This functionality is important given that users can subset the input dataset and GS cohort by sex in the main ‘MethylDetectR’ application. Furthermore, if true age is included, then the application will use this information to subset the sample according to the age slider function on the sidebar panel. If this information is not uploaded, then epigenetic or predicted age will be used to subset the data by age range. In the case where some individuals have true age available and others have missing data, true age will be used for those who have such data and epigenetic age will be used for those without age data in order to the subset the sample. It is strongly recommended that anonymised or pseudonymised IDs are used where possible. For the user’s own convenience in preparing the methylation object, it is recommended that individuals are included as columns and CpG sites as rows. However, this version or a transposed version are accepted and automatically processed by the software. The following features also aid with automation in generating DNAm-based scores for traits:

- Beta values or M values are accepted with the latter converted to beta values by the software.
- Missing methylation values are accepted and mean imputed across input individuals by the software.
- CpG sites that are necessary for the estimation of a trait but are missing in the uploaded dataset are allowed. In this case, each individual in the input dataset receives the mean beta value for a given missing CpG site derived from GS DNAm data. In effect, this gives every individual in the uploaded dataset a constant that brings their score closer to that of the reference sample. In this way, all CpG sites are used for any sample uploaded.

Predicted values or scores for the aforementioned traits can be downloaded as a .csv file which may be uploaded to the main ‘MethylDetectR’ application. Alternatively, an R script is provided to generate these DNAm-based scores if the user does not wish to upload DNAm data or if the DNAm file is too large for upload ( https://doi.org/10.5281/zenodo.4646300 ^26^).

The main ‘MethylDetectR’ application can be described in four modules or panels ( https://shiny.igmm.ed.ac.uk/MethylDetectR/). In the first panel, users can view DNAm-based scores corresponding to various traits for any individual in their input dataset. The user can interactively compare scores for any individual to the remainder of the input dataset. A .csv file may be downloaded which shows percentile ranks for each individual in the uploaded dataset. Percentile ranks are reported for each estimated trait with the exception of the age predictor which is reported in years. If the user uploads an optional binary phenotype file, they can also view the distributions of the DNAm-based scores according to case vs. control status for a given trait of interest. This file should contain IDs of individuals in the methylation file (‘ID’ column) as the first column and the remaining columns may contain any names or traits of interest with individuals coded as ‘0’ for controls and ‘1’ for cases. In the second panel, a plot shows the percentile ranks for a given individual for selected traits. Users can also choose to view the spread of percentile ranks for cases versus controls. Here, the median percentile for cases, along with the first and third quartiles (interquartile range), are plotted for each selected DNAm-based estimated trait. In the third panel, the user can view how different DNAm-based predictors correlate with one another in both the input and GS datasets. In the fourth panel, users can view how DNAm-based scores for age, lifestyle and biochemical traits correlate with phenotypic values for these traits in GS participants (n = 4,450). In each panel, users can subset the samples by age range and sex. In panels 1-3, options to subset the input dataset by case vs. control status are present.

***Development of a DNAm-based predictor.*** Readers are referred to a review on the development of DNAm-based scores and the challenges surrounding their generation ^27^. To develop ‘omics’-based predictors, such as DNAm-based predictors, statistical or machine learning methodologies are commonly applied. In the case of DNAm, the process begins with the quantification of DNAm across individuals using a tissue or cell-type of interest. Many studies focus on blood as it integrates information from various tissues around the body and represents an inexpensive and minimally-invasive approach to gather molecular data. Many cohort studies also have DNA from historic blood samples stored and available to analyse. The collection of saliva and buccal samples is becoming increasingly popular as a relatively low cost and non-invasive method for cohort studies interested in epigenetic epidemiology. The number of CpG sites which are measured depends on the array used but typically includes up to around 800,000 unique sites. Following quantification and quality control, a researcher may wish to study the association between DNAm and a trait of interest, such as smoking status. The average methylation level at a given CpG site across individuals will be correlated with the trait of interest. Methylation levels may be reported between 0-100% for convenience, and a level of 50% means that 50% of cells or DNA molecules within an individual’s sample show methylation at that CpG site. One approach is to correlate each CpG site, in turn, with the trait of interest thereby considering each CpG site in isolation. This is approach is referred to as an epigenome-wide or methylome-wide association study (EWAS or MWAS). Alternatively, methods such as penalised regression can be used to model all CpG sites simultaneously producing parsimonious models that account for correlated features/sites (e.g., least absolute shrinkage and selection operator or LASSO regression) or models that apply small weights to all features/sites (e.g., ridge regression). Elastic net regression is a commonly used intermediate of these two approaches. Correlations among CpGs may arise from sites which lie near each other in a genomic region or from a shared environmental influence; for instance, inhalation of cigarette smoke may affect many CpG sites across different chromosomes. A subset of CpG sites may show a strong relationship with the trait of interest and therefore be informative for predicting the trait in other individuals. The strength of the correlation, or association, is represented by an effect size and provides a weighting for that CpG site’s importance in predicting the trait. In a separate or test group of individuals, predicted values or scores for the trait can be obtained by multiplying DNAm levels at each informative CpG site by their weight derived from statistical analyses. The sum of these products provides a predicted or estimated value for the trait. A statistical transformation can be applied to return DNAm-based scores on the original scale for the trait, such as pack years for smoking. Alternatively, comparison to other individuals may provide meaning to the DNAm-based scores. In any case, predicted values or scores in the test sample may be correlated with true values for a given trait to provide an index of predictive power.

***DNAm-based predictors in ‘MethylDetectR’.*** In ‘MethylDetectR’, we include DNAm-based predictors of chronological age, and six lifestyle and biochemical traits.

Age predictor

The age predictor was developed by Zhang *et al.* using elastic net regression and best linear unbiased prediction (BLUP) ^22, 28, 29^. The two sets of predictors were both built on the same set of 13,566 training samples spread across 14 cohorts. The age predictor was generated using data from individuals with an age range of 2 to 104 years. The elastic net method selected 514 CpG sites as informative for predicting chronological age whereas the BLUP predictor used all CpG sites (319,607 probes). In ‘MethylDetectR’, we apply the elastic net predictor owing to the faster computation and superior performance of this age predictor when compared to the BLUP predictor. The elastic net predictor correlates 0.98 with chronological age (root mean squared error = 2.6 years in GS (n = 4,450) and 2.04 in original publication ^22^). The age predictor is returned in values of years.

Lifestyle and biochemical traits

Previously, we generated ten predictors of lifestyle and biochemical traits in 5,087 individuals within the GS study using LASSO penalised regression ^25, 30^. Ten-fold cross-validation was applied and the mixing parameter (alpha) was set to 1. The lambda value corresponding to the minimum mean cross-validation error was selected and applied to generate the optimal models ^30^. In a test sample consisting of 875 individuals in the Lothian Birth Cohort 1936 study, DNAm-based predictors for four of the traits explained greater than 10% of phenotypic variance in their respective trait. These four traits were alcohol consumption, body mass index, high-density lipoprotein cholesterol and smoking behaviour. There were no phenotypic data available for body fat percentage and waist-to-hip ratio in the Lothian Birth Cohort 1936 study. However, these traits were highly correlated with body mass index in GS (correlation coefficients of 0.6 and 0.4, respectively). Therefore, body fat percentage and waist-to-hip ratio were brought forward to the ‘MethylDetectR’ platform in addition to the four DNAm-based predictors of lifestyle and biochemical traits which demonstrated test R ^2^ statistics of greater than 10% in the Lothian Birth Cohort 1936 sample. Four other traits demonstrated test R ^2^ statistics of less than 10%: educational attainment (2.5%), low-density lipoprotein and remnant cholesterol (0.6%), total cholesterol (2.7%) and total-to-high-density lipoprotein cholesterol ratio (4.5%). These four traits were not brought forward to the ‘MethylDetectR’ platform ^25^.

In the training sample, alcohol intake was assessed in units per week and was only considered in those who reported that their intake was representative of a normal week. A natural log(units + 1) transformation was applied to reduce skewness. For body mass index, extreme values defined as less than 17 kg/m ^2^ or greater than 50 kg/m ^2^ were removed and a natural log transformation was applied. Smoking behaviour was assessed using pack years which is calculated by multiplying the number of packs smoked per day by the number of years the participant has smoked. Current and never smokers were included; ex-smokers were removed owing to complications in adjusting for time since cessation when calculating pack years. To reduce skewness, a natural log(pack years + 1) transformation was applied. In generating the predictors, phenotypes were pre-corrected to remove the influence of age, sex and ancestry using ten genetic principal components. Phenotypic data used to train the predictors were not corrected for cell-type heterogeneity. Further quality control details are available in the original publication ^25^. DNAm values at CpG sites were the independent variables (n = 392,843 CpG sites). CpG sites were filtered to include loci present on both the Illumina EPIC and 450k arrays.

***Version Control.*** We will update ‘MethylDetectR’ every three months to include new DNAm-based predictors of human traits as they are generated by our own group and others. Updates will be managed by Robert F. Hillary or Riccardo E. Marioni. If researchers wish to have their predictors considered for inclusion in ‘MethylDetectR’, please use the corresponding author email address in this manuscript or the contact details available at the ‘MethylDetectR’ website ( https://www.ed.ac.uk/centre-genomic-medicine/research-groups/marioni-group/methyldetectr). The current and historical versions of ‘MethylDetectR’ are available in the Zenodo repository, updated versions will also be made available in this repository ( https://doi.org/10.5281/zenodo.4646300).

### Operation

***Software requirements.*** Both applications are hosted on a secure, patched server hosted at the University of Edinburgh. The applications are developed using Shiny (version 1.4) in R ^31^. The version of R used at the time of ‘MethylDetectR’ development was 3.5.0 ^32^. For ‘MethylDetectR – Calculate Your Scores’, the following R packages were utilised: shinyWidgets (version 0.5.4) ^33^, shinythemes (version 1.1.2) ^34^, data.table (version 1.12) ^35^, shinyalert (version 1) ^36^. For the main ‘MethylDetectR’ application, shinyWidgets (version 0.5.4) ^33^, shinythemes (version 1.1.2) ^34^, data.table (version 1.12) ^35^, shinyalert (version 1) ^36^ were also used, in addition to ggplot2 (version 3.0) ^37^, dplyr (version 0.7) ^38^, forcats (version 0.4.0) ^39^, wesanderson (version 0.3.6) ^40^, shinycssloaders (version 0.2) ^41^, magick (version 2.5.0) ^42^, corrplot (version 0.84) ^43^, ggcorrplot (version 0.1) ^44^ and cowplot (version 0.9.4) ^45^. Scripts for implementing both applications are available at: https://doi.org/10.5281/zenodo.4646300 ^26^. The script has been designed to ensure automatic installation of missing CRAN packages which are necessary for the operation of ‘MethylDetectR’.

**Figure 1.**
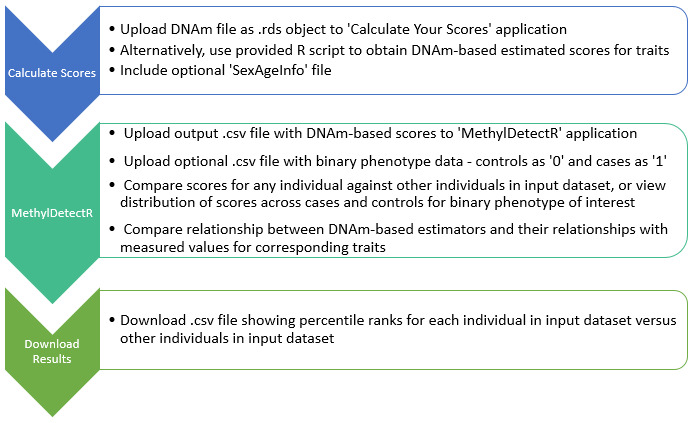
Overview of workflow in ‘MethylDetectR’ platform. Users upload DNAm data to a secure application named ‘MethylDetectR – Calculate Your Scores’ or use a provided R script to locally generate DNAm-based values for age and a variety of lifestyle and biochemical traits. DNAm-derived scores are submitted to the ‘MethylDetectR’ application to view these scores, interactively compare scores within the input sample and optionally view the distribution of scores across cases and controls for uploaded binary phenotypes of interest. CSV files are available to download showing percentile ranks for individuals in the input dataset against other individuals in the uploaded dataset.

***Overview of workflow.*** The main components of the platform are outlined in the Implementation section and the associated workflow is graphically depicted in Figure 1.

## Use cases

### MethylDetectR – Calculate Your Scores

The user can upload a DNAm file as an R object (.rds file) to the ‘MethylDetectR – Calculate Your Scores’ application. Beta values or M values may be used. If M values are detected, these are converted to beta values. It is recommended that individuals are included as columns and CpG sites (derived from Illumina arrays) are included as rows and that file sizes of no greater than 500 MB are uploaded. The application details general information on the platform, as well as information on how to format files and links to all elements of the platform. To make DNAm files smaller prior to upload, users can access a file called ‘Truncate_to_these_CpG.csv’ which is available in the Zenodo repository. This file allows users to subset CpGs measured in their dataset to those used in ‘MethylDetectR – Calculate Your Scores’ making files considerably smaller. A ‘SexAgeInfo’ file may also be uploaded as a .csv file so that data pertaining to the ages and sex of the input individuals are included in the output file along with DNAm-based values for age (epigenetic age), lifestyle and biochemical traits. This file should include a column corresponding to the IDs of individuals in the methylation file (‘ID’ column), a column that lists the sex of these individuals written as ‘Male’ or ‘Female’ or ‘NA’ (‘Sex’ column) and a column that reports the chronological or true ages of individuals (‘Age’ column). Alternatively, the user can merge this information into their DNAm score file after running the ‘MethylDetectR – Calculate Your Scores’ step. Examples for the DNAm and ‘SexAgeInfo’ files are available at: https://doi.org/10.5281/zenodo.4646300 ^26^.

**Figure 2.**
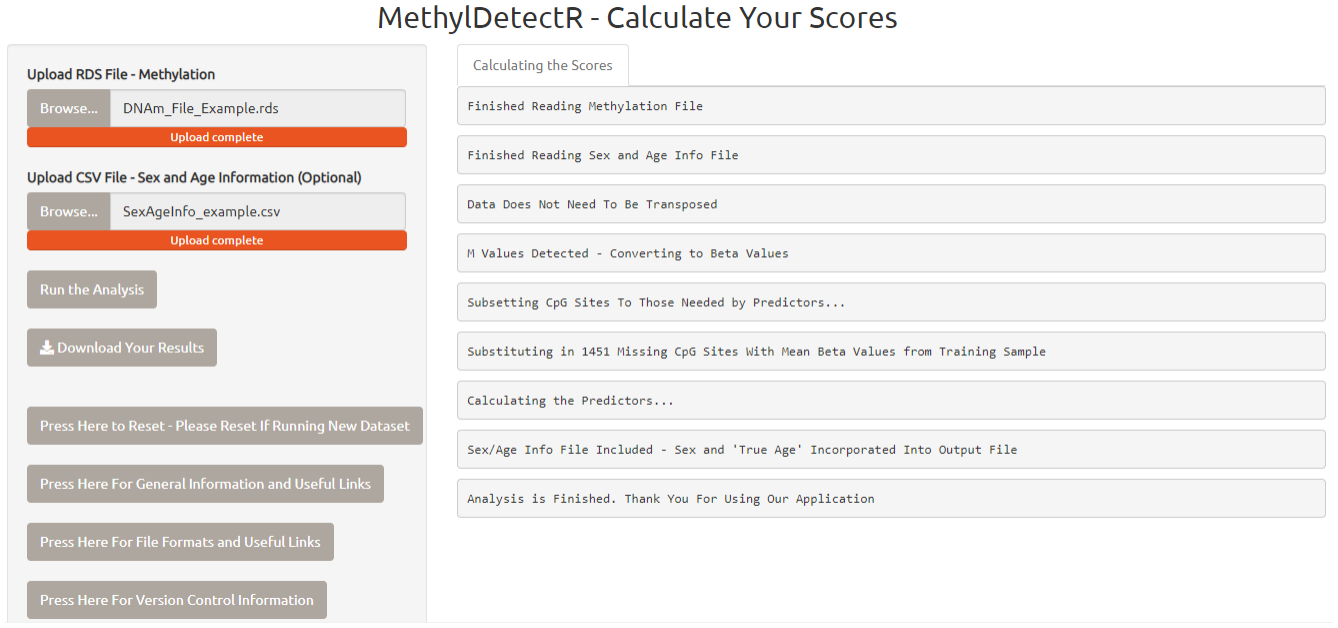
MethylDetectR – Calculate Your Scores Application. An example session for the ‘MethylDetectR – Calculate Your Scores’ application. In this case, a DNAm dataset, stored as an R file .rds object, has been uploaded along with the optional ‘SexAgeInfo’.csv file. A log output has been generated for the user detailing quality control steps which have been carried out in the calculation of DNAm-based predictors. For instance, M values were uploaded and converted to beta values. The resultant output file can be downloaded as a .csv file for upload to the main ‘MethylDetectR’ application.

The software automatically generates DNAm-based scores for every individual in the dataset (.csv file) and a report for the user is printed on the application detailing quality control steps carried out during the calculation process. For example, the report informs the user whether or not the data had to be transposed, or if M values were converted to beta values ( Figure 2).

Alternatively, the user can download an R script to locally generate DNAm-based scores for the traits. The DNAm object is annotated as ‘data’ and the ‘SexAgeInfo’ input is annotated as ‘sexageinfo’ ( https://doi.org/10.5281/zenodo.4646300 ^26^). In either case, an output .csv file is generated containing DNAm-based scores or values for each trait and for every individual in the input dataset. This output file should be uploaded to the main ‘MethylDetectR’ application. An example output .csv file showing the correct column names and file structure is available at: https://doi.org/10.5281/zenodo.4646300 ^26^.

### MethylDetectR

***Panel 1.*** The output file from either the ‘MethylDetectR – Calculate Your Scores’ application or a publicly available script should be uploaded to the main ‘MethylDetectR’ application. Incorrectly assigned column names will be reported to the user, as will files with no individuals or files with non-numeric values. A timeout is triggered following three minutes of inactivity. All panels contain information on the data shown in the panel, and Panel 1 details information on how to format files. Links to all other elements of the platform are shown in each panel via ‘info’ buttons located in the sidebar panels.

**Figure 3.**
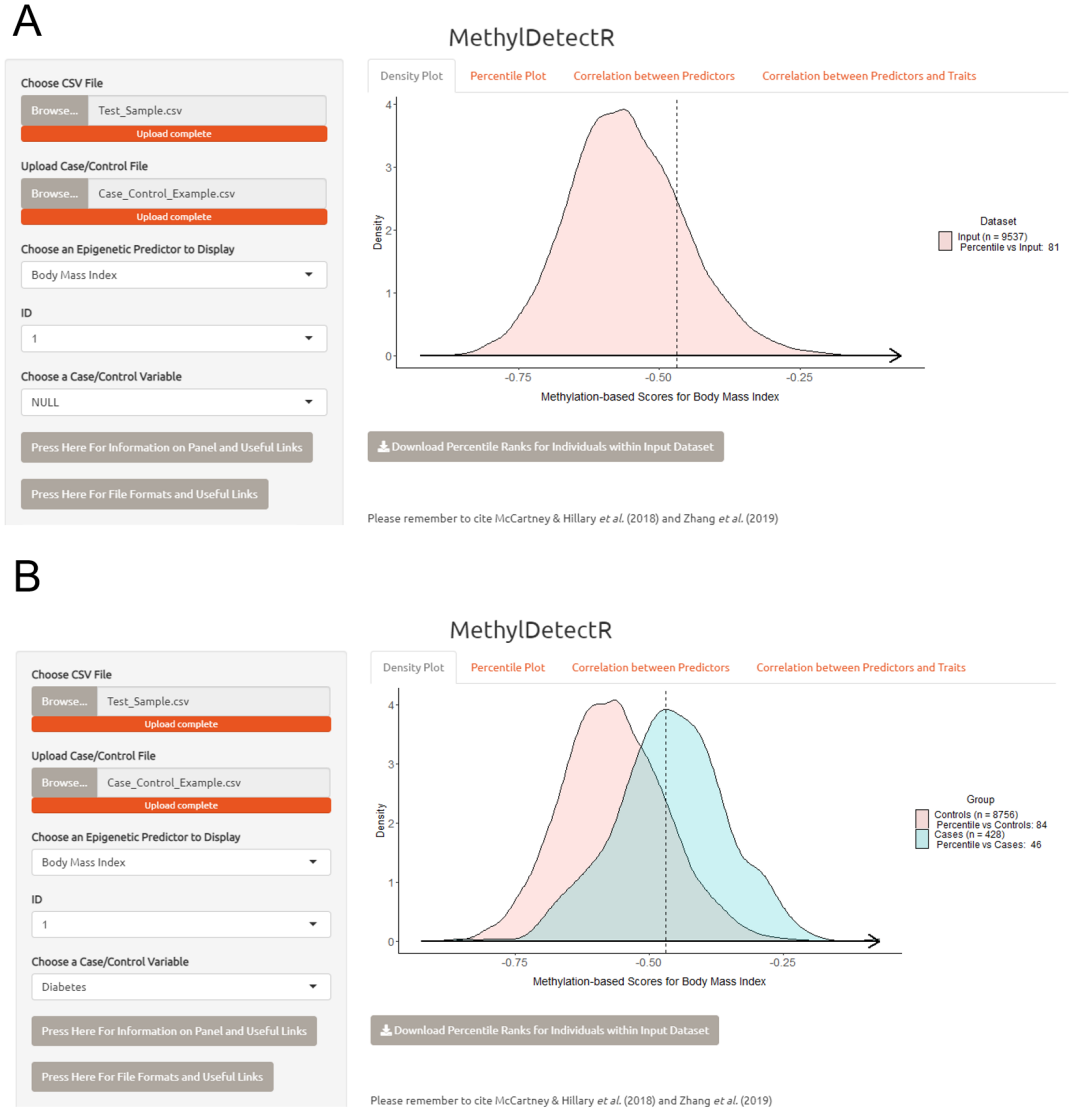
MethylDetectR Application – Panel 1. ( **A**) In the first panel, users can select a variable of interest and view how the methylation-based estimate for a chosen individual in the input dataset compares against the remainder of the input dataset (pink). ( **B**) The user can subset the scores according to case vs. control status for an uploaded binary phenotype of interest, such as disease status. Here, the distributions of DNAm-based scores for body mass index are plotted for diabetes cases (blue) and controls (pink). Percentile ranks for all individuals in the input dataset when compared against other members of the input dataset may be downloaded as a .csv file in this panel. Percentile ranks are reported for all traits in the output file with the exception of age which is reported in years.

The first panel allows users to choose a predictor of interest and view how a selected individual in the input dataset ranks against the remainder of the input dataset (in pink) in the context of that predictor ( Figure 3A). Alternatively, if the user uploads an optional file with binary phenotype information, then users can also subset the data by case vs. control status. In Figure 3B, the user can view where a selected individual’s DNAm-based score for body mass index lies along the sample subset by controls (in pink) and cases (in blue) for diabetes. The user can subset to different age ranges and sex in order to see how the selected individual would compare to the truncated sample selection. Users can also download the percentile ranks for every individual in the input dataset when compared against all other individuals in the dataset. Percentile ranks are available for each trait with the exception of age which is reported in years.

**Figure 4.**
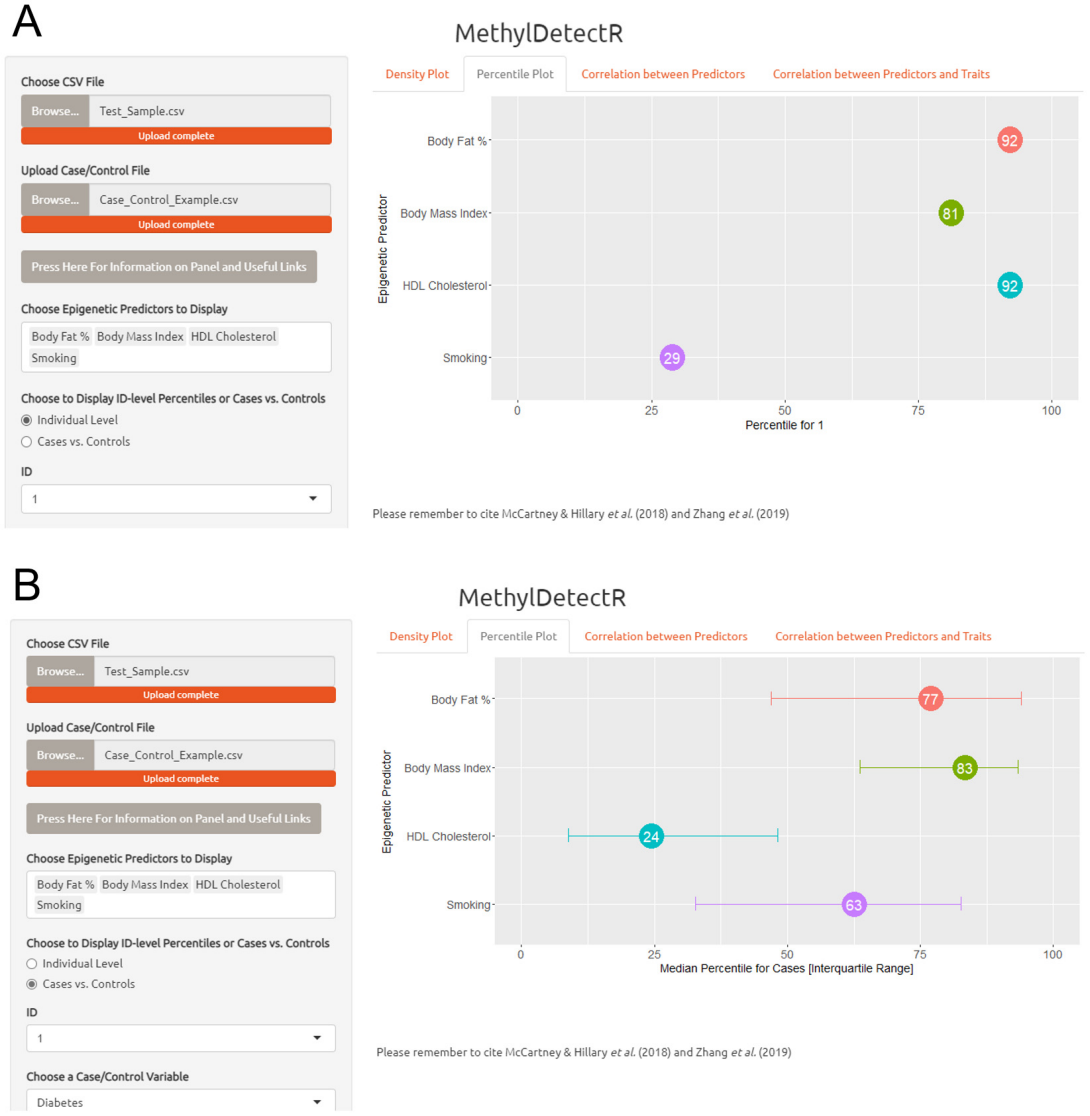
MethylDetectR Application – Panel 2. ( **A**) In the second panel, users can select multiple predictors of interest and simultaneously view the percentile rank for a selected individual in relation to these traits when compared against the remainder of the uploaded dataset. The percentile ranks dynamically update according to the selected age range and sex. ( **B**) The median percentile ranks for cases are plotted for selected traits of interest. The number shown in the circle reflects the median percentile, and the interquartile range of percentile ranks for cases is shown with the horizontal lines extending from the circle. Here, the median percentile ranks with respect to a number of physical traits are shown for cases of diabetes.

***Panel 2.*** In the second panel, users may select multiple traits in order to simultaneously view the percentile ranks for a selected individual in the input dataset when compared against other individuals in the sample ( Figure 4A). Furthermore, the user can view how percentile ranks for a given trait vary according to cases and controls for a selected binary phenotype. In Figure 4B, the median percentile for diabetes cases along with the interquartile range (first to third quartile) are plotted for multiple traits, such as body mass index and body fat percentage. Again, the user can use a sidebar functionality to subset by age range and sex.

**Figure 5.**
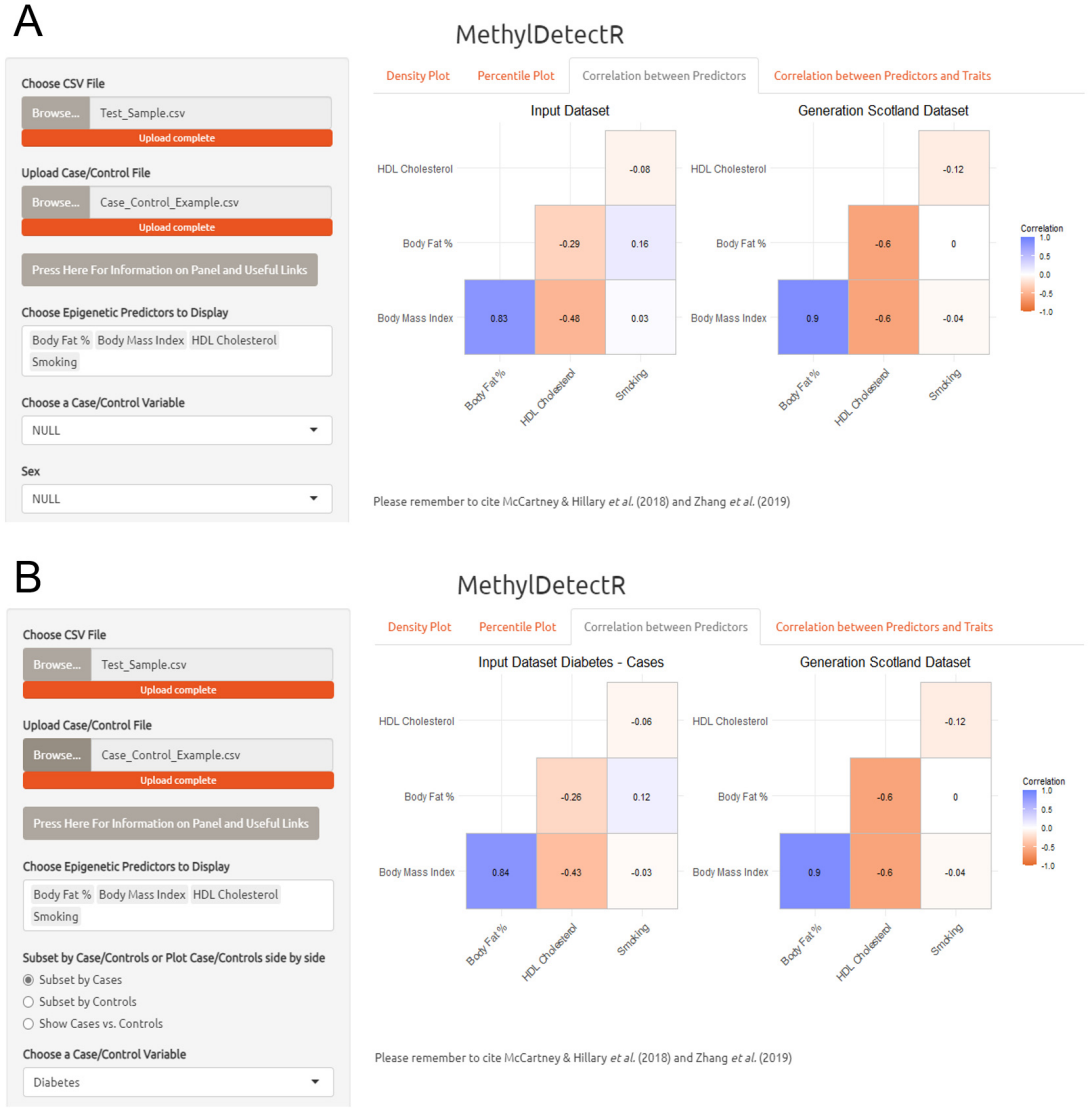
MethylDetectR Application – Panel 3. ( **A**) In the third panel, users can select multiple predictors of interest and simultaneously view the interrelationships between these variables of interest in both the input dataset and a reference sample - GS (n = 4,450). ( **B**) The user can subset the input dataset according to cases or controls, or choose to view the data structure for cases and controls side by side. The correlation coefficients are updated according to the selected age range and sex.

***Panel 3.*** In the third panel, users can select multiple DNAm-based predictors and view how they correlate with one another in order to visualise their interrelationships and the underlying data structure. This is represented for both the input and GS datasets ( Figure 5A). Furthermore, the correlations are updated according to the selected age range and sex. The user can also subset the input dataset to cases, controls or choose to visualise correlation data for cases and controls in the input dataset alongside each other ( Figure 5B). 

**Figure 6.**
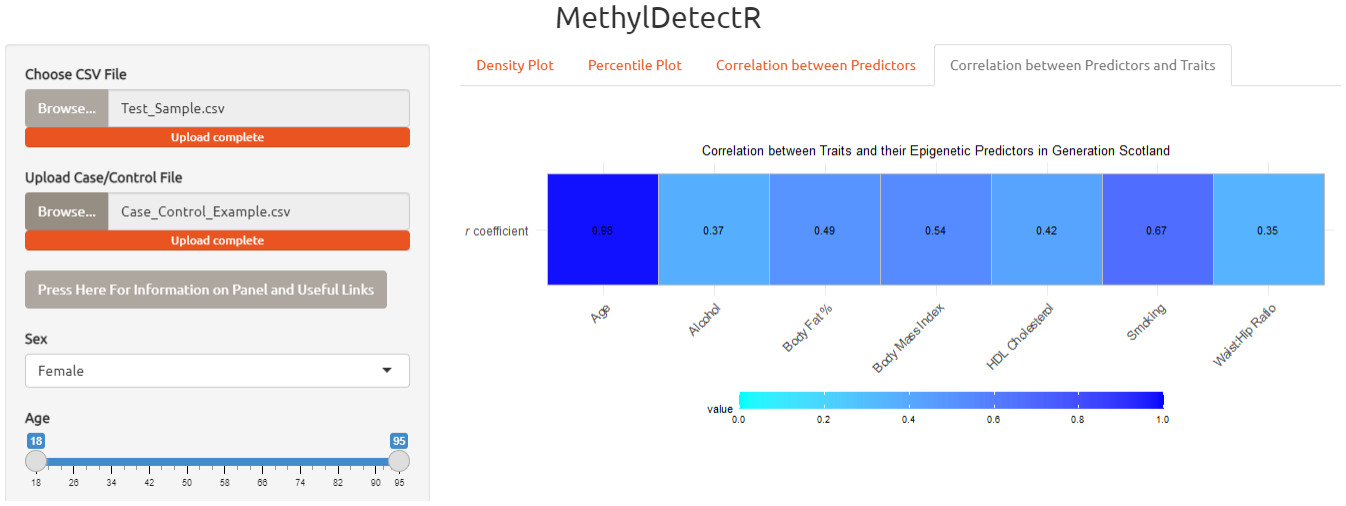
MethylDetectR Application – Panel 4. In the fourth panel, users can view how age, lifestyle traits and high-density lipoprotein (HDL) cholesterol relate to phenotypic or measured values in the GS reference sample (n = 4,450). Correlation coefficients for age, lifestyle traits and HDL cholesterol are updated according to the selected age range and sex. HDL (high-density lipoprotein).

***Panel 4.*** In the fourth and final panel, users can view how well the DNAm-based predictors for age, lifestyle traits and HDL cholesterol correlate with actual values of their respective traits in GS ( Figure 6). In this final panel, users can also subset by age range and sex to view how the performance of the predictors varies according to the truncated reference dataset.

## Discussion

We have created and implemented the first publicly available online translational platform for methylation-based health profiling. The platform includes a wide variety of traits which are estimated from large-scale DNAm data. These include chronological age, lifestyle traits and biochemical data thereby providing an automatic and comprehensive estimate of individual health profiles from a single blood draw. Users can interactively view how well DNAm-based estimators for various traits perform at an individual level and how DNAm-based estimators stratify according to case and control status for binary phenotypes of interest. The ‘MethylDetectR’ platform communicates key messages surrounding the development and present limitations of DNAm-based health profiling to the wider research community and public. This is achieved by including ‘info’ buttons in the sidebar panels of each application that lead to important information for interpreting the presented results, key limitations and general information on DNAm-based scores. Furthermore, the platform is designed to ensure the highest level of data security and safety and is publicly available with open source code and example input and output files. We will continue to update ‘MethylDetectR’ every three months with the aim of including new DNAm-based predictors of human traits when they come available.

DNAm-based predictors can integrate biological and environmental information to provide important indices of an individual’s health status and well-being. These predictors must display high degrees of sensitivity and specificity in order to accurately distinguish individuals on trajectories toward disease and adverse clinical endpoints from those who will remain healthy in a given clinical context. Currently, the DNAm-based predictors in ‘MethylDetectR’ cannot make consistently accurate predictions at an individual level and therefore cannot yet be reliably applied in a diagnostic or forensic context. Highly-accurate DNAm-based scores can aid in research environments as they may provide more accurate information than self-report data ^20^. For instance, the DNAm-based predictor of smoking can provide a more accurate profile of smoking history than responses from participants in questionnaires. Further, the use of DNAm-based scores for a variety of human traits can proxy many phenotypes such as biochemical and lifestyle traits using a single blood draw. Together, these data can help researchers determine relationships between putative risk factors and important health outcomes, and aid in patient stratification paradigms. Further, ‘MethylDetectR’ serves as an important translational tool showing an interactive, demo version of the platform and substantial information within each application regarding the interpretation and limitations of DNAm-based predictors. As these predictors become refined, they may be of clinical value. For instance, a blood-based DNAm test was recently developed that could detect five separate types of cancer up to four years before conventional diagnosis. The assay measured circulating tumour DNA methylation and predicted disease in 88% of post-diagnosis patients, with a specificity of 96% ^19^. 

Distributions of DNAm values and subsequent DNAm-based scores may vary across different methylation datasets. In relation to the biochemical and lifestyle traits, the predictors were generated using an adult sample of individuals with European ancestry. Therefore, it is possible that the predictors may not be generalisable to datasets comprising different age ranges, such as cohorts of children, and individuals with different ancestries. Differences between datasets may also arise from biological differences, for example cases for a given disease may have altered DNAm values for a number of probes relative to controls, or result from technical or normalisation differences. As a result, DNAm-based scores may vary greatly across datasets and projecting an individual onto a reference sample to view where their DNAm-based score would lie along the reference sample is therefore challenging. Future work will focus on developing methods which can appropriately account for variability across datasets and allow for a projection of individuals onto disparate DNAm samples or datasets.

Increased sample sizes through recruitment, consortia or meta-analyses may allow for more sensitive or specific DNAm-based predictors. Advancements in statistical and machine learning approaches used to generate such predictors will also allow for greater accuracy in predicting human traits and health ^23^. Furthermore, if the outcomes on which the predictors are trained are inaccurate or possess lots of noise, then the predictors themselves will perform poorly in identifying individuals at risk of disease. Therefore, advancements in understanding disease biology and ways to diagnose or stratify different diseases will help to create well-defined outcomes on which predictors can be trained. This is expected to improve their ability in predicting important health and clinical outcomes. However, stringent ethical frameworks are also necessitated prior to widespread application of molecular-based health profiling in health and forensic contexts ^46^.

DNAm-based predictors represent one avenue within molecular-based health profiling. Genetics-based predictors of human traits may correlate well with true values for traits, such as human height ^47^. However, genetic predictors of disease may often fail to accuracy classify individuals by disease status ^48^. Additionally, other ‘omics’ data have been explored in order to predict human traits or disease. For example, a proteomic signature of age correlates 0.94 with chronological age ^49^. Plasma protein-based predictors of disease states, including dementia and cancer, have been explored ^50– 52^. Lipid-based predictors of human traits have also been developed using plasma samples ^53, 54^. Complex and common disease states are multifactorial conditions. Therefore, it is likely that composite predictors using various lines of ‘omics’ data may allow for greater accuracy in predicting disease risk and outcomes when compared to using one line of evidence alone. Furthermore, the incorporation of ‘omics’ data with clinical or demographic data could provide even more refined predictors of human health and disease ^48^.

## Conclusions

Our platform provides an important translational tool which communicates state-of-the-art developments in relation to DNAm-based predictors of human traits and health. The ‘MethylDetectR’ platform also represents a research tool for the convenient and secure generation of DNAm-estimated traits for use in clinical and population studies. Importantly, our platform highlights the applicability and limitations surrounding such predictors prior to their potential deployment in clinical assessment and management paradigms. 

**Reproducibility:** All relevant code is available at: https://doi.org/10.5281/zenodo.4646300 ^26^. The following Research Resource Identifiers (RRIDs) have been generated for ‘MethylDetectR – Calculate Your Scores’, RRID: SCR_018972, and ‘MethylDetectR’, RRID: SCR_018973. The limitations surrounding the resultant datasets are that the predictors may work well for risk stratification relative to others in the dataset, but may fail to accurately predict trait information at an individual level.

## Data availability

### Underlying data

The underlying methylation and phenotypic data used to generate the original predictors cannot be made available due to the data containing information that could compromise participant consent and confidentiality. According to the terms of consent for GS participants, access to data must be reviewed by the GS Access Committee. Applications should be made to access@generationscotland.org. Lothian Birth Cohort 1936 data can be requested from the Lothian Birth Cohort 1936 research team, following completion of a data request application. More information can be found online ( http://www.lothianbirthcohort.ed.ac.uk/content/collaboration). 

### Extended data

Zenodo: MethylDetectR - A Translational Tool for Methylation-Based Health Profiling, https://doi.org/10.5281/zenodo.4646300 ^26^

This project contains the following extended data:

- DNAm_File_Example.rds. (Example DNAm input file for upload to ‘MethylDetectR – Calculate Your Scores’.)
- SexAgeInfo_example.csv. (Example optional ‘SexAgeInfo’ input file for upload to ‘MethylDetectR – Calculate Your Scores’.)
- Truncate_to_these_CpGs.csv. (File for truncating DNAm input file to CpG sites used in ‘MethylDetectR – Calculate Your Scores’.)
- MethylDetectR_Test_for_Upload.csv. (Example input file for upload to main ‘MethylDetectR’ application.)
- MethylDetectR_Case_Control_Example.csv. (Example binary phenotype .csv file with controls coded as ‘0’ and cases coded as ‘1’.)
- Script_For_User_To_Generate_Scores.R. (R script for user to locally generate DNAm-based estimates of human traits for upload to ‘MethylDetectR’ application.)
- Predictors_Shiny_by_Groups.csv. (Associated file for use in ‘Script_For_User_To_Generate_Scores.R.)

Data are available under the terms of the Creative Commons Attribution 4.0 International license (CC-BY 4.0). 

## Software availability

Zenodo: MethylDetectR - A Translational Tool for Methylation-Based Health Profiling, https://doi.org/10.5281/zenodo.4646300 ^26^.

This project contains the following scripts:

- MethylDetectR – Calculate Your Scores.R (R script for running of ‘MethylDetectR – Calculate Your Scores’ application.)
- MethylDetectR.R. (R script for running of ‘MethylDetectR’ application.)

Data are available under the terms of the Creative Commons Attribution 4.0 International license (CC-BY 4.0). 

### Reporting guidelines

Open Science Framework: TRIPOD checklist for ‘MethylDetectR - a translational platform for methylation-based health profiling’, https://doi.org/10.17605/OSF.IO/3MGJT ^55^

The TRIPOD checklist is available at: https://www.tripod-statement.org/wp-content/uploads/2020/01/Tripod-Checlist-Prediction-Model-Development.pdf ^56^.

Data are available under the terms of the Creative Commons Attribution 4.0 International license (CC-BY 4.0). 
