# Supplementary material for: MethylDetectR: a software for methylation-based health profiling
Source: Wellcome Open Res. 2021 Apr 13;5:283. Originally published 2020 Dec 7. [Version 2] doi: 10.12688/wellcomeopenres.16458.2 (PMC8080939; doi:10.12688/wellcomeopenres.16458.2)
Supplement: Peer review history

# Peer review: Reviewer response for version 2

**DOI:** 10.21956/wellcomeopenres.18504.r43463

**Published:** 2021-04-27

**Authors:** Charlotte Cecil

**Affiliations:** 1 Department of Child and Adolescent Psychiatry, Erasmus MC, Rotterdam, The Netherlands; 2 Department of Epidemiology, Erasmus MC, Rotterdam, The Netherlands; 3 Molecular Epidemiology, Department of Biomedical Data Sciences, Leiden University Medical Center, Leiden, The Netherlands; 4 Institute of Psychiatry, Psychology & Neuroscience, King's College London, London, UK

The authors have done an excellent job at revising the manuscript and online application in light of the reviewer comments. I particularly commend the authors for taking the time to amend the app by providing information that will help users to contextualize and interpret the scores - I believe that it is now much improved and  have no further comments.

Are the conclusions about the tool and its performance adequately supported by the findings presented in the article?

Partly

Is the rationale for developing the new software tool clearly explained?

Yes

Is the description of the software tool technically sound?

Yes

Are sufficient details of the code, methods and analysis (if applicable) provided to allow replication of the software development and its use by others?

Yes

Is sufficient information provided to allow interpretation of the expected output datasets and any results generated using the tool?

No

Reviewer Expertise:

Epigenetic epidemiology; psychiatric epidemiology; child development; developmental psychopathology; early life stress.

I confirm that I have read this submission and believe that I have an appropriate level of expertise to confirm that it is of an acceptable scientific standard.

# Peer review: Reviewer response for version 2

**DOI:** 10.21956/wellcomeopenres.18504.r43464

**Published:** 2021-04-13

**Authors:** Gemma Sharp

**Affiliations:** 1 MRC Integrative Epidemiology Unit, Population Health Sciences, University of Bristol, Bristol, UK

The authors have updated the manuscript and I am satisfied with the amendments.

Are the conclusions about the tool and its performance adequately supported by the findings presented in the article?

Yes

Is the rationale for developing the new software tool clearly explained?

Yes

Is the description of the software tool technically sound?

Yes

Are sufficient details of the code, methods and analysis (if applicable) provided to allow replication of the software development and its use by others?

Yes

Is sufficient information provided to allow interpretation of the expected output datasets and any results generated using the tool?

Partly

Reviewer Expertise:

Epigenetic epidemiology, R, Shiny.

I confirm that I have read this submission and believe that I have an appropriate level of expertise to confirm that it is of an acceptable scientific standard.

# Peer review: Reviewer response for version 1

**DOI:** 10.21956/wellcomeopenres.18118.r42132

**Published:** 2021-02-16

**Authors:** Charlotte Cecil

**Affiliations:** 1 Department of Child and Adolescent Psychiatry, Erasmus MC, Rotterdam, The Netherlands; 2 Department of Epidemiology, Erasmus MC, Rotterdam, The Netherlands; 3 Molecular Epidemiology, Department of Biomedical Data Sciences, Leiden University Medical Center, Leiden, The Netherlands; 4 Institute of Psychiatry, Psychology & Neuroscience, King's College London, London, UK

In the present study, the authors present a new publicly available online platform for methylation-based health profiling. The manuscript is clear, well written and addresses an important emerging topic in population epigenetics. The online platform enables the calculation of multiple DNA methylation (DNAm) scores for age, lifestyle factors and protein levels related to neurology or inflammation. Based on these scores, users are able to view percentile ranks for specific individuals as compared to others in the input sample, with the option to subset by case-control status, sex and age. Users are also able to estimate a broader health profile for specific individuals by displaying percentiles for multiple DNAm scores simultaneously. Finally, users can check correlations between their predicted DNAm scores compared to those in the Generation Scotland dataset, as well as correlations between predicted DNAm scores and actual measured traits in Generation Scotland. Overall, I think this is an excellent contribution to the field and an important first step for moving methylation-based predictors into translational applications. I am concerned however, that no practical information is provided, especially within the online platform, to aid users in the correct interpretation of the scores themselves, and the findings generated from input datasets. If the platform is to reach widespread use and fulfil its translational potential, I would anticipate that part of the users may not have a research background and many may not read this manuscript in detail before utilizing the platform. As such, I think it is essential to add information within the online tool to guide users and ensure that scores are interpreted adequately with full transparency on limitations (e.g. applicability to other age ranges, populations, error around DNAm estimates, meaning of different percentile ranks and how those should be interpreted etc.). I would also like to see in the manuscript a more detailed discussion of potential uses for this platform in different contexts, using practical examples (e.g. screening and risk prediction in research and clinical settings). I believe these steps are necessary in order to ensure that the platform is adequately used without DNAm scores being overly- or incorrectly-interpreted, especially if the hope is that such a platform will be utilized in a clinical context. Specific comments are listed below.

- How 'live' is this online resource? Given the fast-paced developments in population epigenetic methods and prediction tools, how regularly will the resource be updated to take stock of these developments (both in terms of the actual calculation of methylation-based scores, as well as the information provided regarding applicability and limitations)?
- Methods – implementation
  - The authors provide a link to their online interface for calculating DNAm scores. I would anticipate that, at least for research use, many studies would not be allowed to upload individual level DNAm data due to data protection policies, even when anonymized (some institutions have concerns that omics level data can never be truly anonymized), so it is useful that scripts are also provided to run the analyses locally.
  - Is it possible to upload potential covariates, such as cell-type, batch, ancestry etc.? How were these factors, especially cell-type heterogeneity, taken into account in the development of MethylDetecR DNAm scores?
  - Is this tool adequate for input datasets containing samples who may differ in characteristics from those upon which the algorithms were based (e.g. datasets of different ancestry)? Further, could the tool be utilized on child populations, where certain but not all scores would be applicable (e.g. BMI but not alcohol), if the algorithms were developed on adults? And regarding protein levels, how significant is the fact that these scores were developed from a sample of adults in old age? Can we assume that the same relationships between DNAm and protein levels hold across different life stages?
- Demo
  - It would help to add information in the demo about how to interpret the different modules (e.g. a walkthrough, or pop-up bubbles), for example:
    - **First module** how should a user (especially one who may not have a research background) interpret the density plots, what this means when stratified by case-control status, and how to evaluate a given individual’s score within these plots? For example, with regards to the smoking DNAm score, what should a user conclude if an individual of interest scores on the 75 ^th^ percentile overall, and specifically on the 99 ^th^ percentile within controls and 14 ^th^ percentile within cases when stratified by smoker case-control status? Should the user interpret this as a sign that the individual in question is (probably) a smoker or ex-smoker? (p.s. I see later in the manuscript that ex-smokers were removed from the data upon which the prediction was based, which makes the above example all the more relevant).
    - **Second module** how should users interpret the percentile ranks for multiple traits? I would expect users, especially for more clinical applications, to look for a particular threshold with which to guide their interpretation. For example, should anyone with scores above a certain percentile (e.g. 50 ^th^, 75 ^th^ or higher) be considered to have a risky profile (e.g. in terms of DNAm signatures for smoking, body fat etc.)? With regards to the protein DNAm scores, how should a user interpret an individual scoring for example on the 27 ^th^ percentile for IL6, but the 80 ^th^ percentile for TNF.alpha? What implications would this have for making assessments (and thus also potential recommendations) regarding this person’s inflammation profile?
      - On a related note, is there a way to help users gauge how ‘reliable’ these different scores are? Some DNAm predictors (e.g. smoking) are more reliable and accurate than others, could this be indicated in the platform e.g. by visualizing this in the plots or adding text to aid interpretation (i.e. to what extent could a user interpret the percentiles for different scores with confidence)? I see in page 10 that error bars can be displayed, but (a) I do not see where the option to visualize these is in the online tool and this is also not explained in the manuscript; (b) I am assuming that these error bars are related to the input data, so a bit different from the point of indicating how good the scores themselves are. I assume that this can be partly gauged by the information on correlations in the fourth panel, although this includes only a selection of the DNAm scores.
    - **Third panel** how should users interpret differences in correlations between their input dataset and the reference GS dataset? What are the implications of these differences for interpreting their results (e.g. does it mean that scores should be interpreted with more caution or has no bearing on this)? For example, if the correlation between alcohol and smoking DNAm scores in the input dataset is 0.40, but in GS it is only 0.20, what does this mean for the user?
- In the discussion, the authors state that ‘ *the MethylDetecR platform communicates key messages surrounding the development and present limitations of DNAm-based health profiling* *to the wider research community and public’* - where is this information provided? I cannot see it either in the Demo or Calculate your Scores platforms. This should be provided in the Demo, as the first ‘port of entry’ users should be using to get familiarized with the platform (and there should be a notice on the Calculate your Scores platform to first check out the Demo, for an example of correct use). This is a good place to walk users through the different functionalities/panels of the platform, and for each panel, to provide key information on interpretation (e.g. using case examples – “here is Joe, he is estimated to be xx years old and score within the xx percentile for yy variable. Based on this, we can conclude that xxx. Limitations to keep in mind are xxx. This information could be used for xxx applications”).
- Similarly, in the conclusion, the authors state that “ *our platform provides an important translational tool which communicates state-of-the-art developments in relation to DNAm-based predictors of human traits and health…* *and* *highlights the applicability and limitations surrounding such predictors prior to their potential deployment in clinical assessment and management paradigms*.” While the platform allows to calculate state-of-the-art scores, I cannot see anything related to communication of its uses and limitations.
- Generally, the manuscript refers to a lot of different files and links (e.g. the Demo, the actual platform, all the Zenodo materials). This relies on the idea that users in future will always refer to the manuscript and read it in detail before using the platform. I would suggest having an easy to find, centralized repository for all of these materials, or alternatively, to add the various links in the Demo/Calculate your Scores platforms (which should be linked somehow).
- The discussion is missing a section on the research and clinical applications of this platform. Can the authors provide concrete examples of where and how this information could be useful?
- Minor points
  - P.3: “ *In contrast to genetics, these molecular modifications are dynamic, tissue-specific and reversible*” – would qualify this sentence a little bit. While it is correct that, overall, DNAm differ largely across tissues, some sites do show strong correspondence across multiple tissues. Similarly, it is unclear whether all DNAm changes are reversible (e.g. some smoking-related DNAm alterations persisting post-cessation).
  - P. 3: “ *Furthermore, there has been a rapid increase in the commercialisation and scalability of DNAm assays for direct-to-consumer use or for use in clinical, research or industrial settings*.” Can the authors add references or include a couple of examples?
  - P.3: “ *Blood DNAm data …provides a good index of the overall health status of the body*.” Can the authors add a reference to support this?
  - P. 4: *“Lastly, the user can subset the reference samples by sex, age range or case vs. control status.”* Case-control status on what variables?
  - P. 5: https://www.ed.ac.uk/centre-genomic-medicine/research-groups/
  - marioni-group/methyldetectr the link provided has two attachments, which appear to contain the same information: MethylDetectR Participant Information Sheet and MethylDetectR Participant Consent Statement
  - P. 5: in the explanation of methylation risk scores, the authors could point readers to a new review on the topic, including strengths and challenges of this approach (Huls & Czamara, 2020 *Epigenetics*) ^1^.
  - P. 6: Age predictor - can the authors specify the age range of the discovery data upon which the predictor was based?
  - P. 6: Can the author provide a reference/specify how cutoffs were selected for their protein analyses (e.g. for training-test ratio, *r *and *p *thresholds etc.).

Are the conclusions about the tool and its performance adequately supported by the findings presented in the article?

Partly

Is the rationale for developing the new software tool clearly explained?

Yes

Is the description of the software tool technically sound?

Yes

Are sufficient details of the code, methods and analysis (if applicable) provided to allow replication of the software development and its use by others?

Yes

Is sufficient information provided to allow interpretation of the expected output datasets and any results generated using the tool?

No

Reviewer Expertise:

Epigenetic epidemiology; psychiatric epidemiology; child development; developmental psychopathology; early life stress.

I confirm that I have read this submission and believe that I have an appropriate level of expertise to confirm that it is of an acceptable scientific standard, however I have significant reservations, as outlined above.

## References

[1] : Methodological challenges in constructing DNA methylation risk scores. *Epigenetics*.15(1-2) : 10.1080/15592294.2019.1644879 1-11 10.1080/15592294.2019.1644879 31318318PMC6961658

# Response

**Published:** 2021-03-30

**Authors:** Robert Hillary

**Affiliations:** University of Edinburgh, UK

**Comment 1:** I am concerned, however, that no practical information is provided, especially within the online platform, to aid users in the correct interpretation of the scores themselves, and the findings generated from input datasets. If the platform is to reach widespread use and fulfil its translational potential, I would anticipate that part of the users may not have a research background and many may not read this manuscript in detail before utilizing the platform. As such, I think it is essential to add information within the online tool to guide users and ensure that scores are interpreted adequately with full transparency on limitations (e.g. applicability to other age ranges, populations, error around DNAm estimates, meaning of different percentile ranks and how those should be interpreted etc.).

*Response: We thank the reviewer for their excellent suggestions towards improving the platform. We agree that the applications would benefit from more general information on what the predictors reflect and what we can conclude from them in light of limitations. This would achieve an appropriate balance between its applicability for expert and non-expert users alike. As a result, we have added ‘info’ action buttons on each panel of the applications to reveal important information pertaining to the DNAm-based scores and their interpretation. Further, we include links in each application to all other elements of the platform i.e. the other applications, the website, the paper and the Zenodo repository.*

**Comment 2: **I would also like to see in the manuscript a more detailed discussion of potential uses for this platform in different contexts, using practical examples (e.g. screening and risk prediction in research and clinical settings). I believe these steps are necessary in order to ensure that the platform is adequately used without DNAm scores being overly- or incorrectly-interpreted, especially if the hope is that such a platform will be utilized in a clinical context.

*Response: We thank the reviewer for highlighting the need to include more information regarding potential applications of the DNAm-based scores in ‘MethylDetectR’ in research and clinical settings and the appropriate interpretation of their potential utility. To address this, we have amended Paragraph 2 in the ‘Discussion’ section to include the following:*

*“Currently, the DNAm-based predictors in ‘MethylDetectR’ cannot make consistently accurate predictions at an individual level and therefore cannot yet be reliably applied in a diagnostic or forensic context. Highly-accurate DNAm-based scores can aid in research environments as they may provide more accurate information than self-report data* *(6)* *. For instance, the DNAm-based predictor of smoking can provide a more accurate profile of smoking history than responses from participants in questionnaires. Further, the use of DNAm-based scores for a variety of human traits can proxy many phenotypes such as biochemical and lifestyle traits using a single blood draw. Together, these data can help researchers determine relationships between putative risk factors and important health outcomes, and aid in patient stratification paradigms. Further, ‘MethylDetectR’ serves as an important translational tool showing an interactive, demo version of the platform and substantial information within each application regarding the interpretation and limitations of DNAm-based predictors. As these predictors become refined, they may be of clinical value. For instance, a blood-based DNAm test was recently developed that could detect five separate types of cancer up to four years before conventional diagnosis. The assay measured circulating tumour DNA methylation and predicted disease in 88% of post-diagnosis patients, with a specificity of 96%”.*

**Comment 3:** How 'live' is this online resource? Given the fast-paced developments in population epigenetic methods and prediction tools, how regularly will the resource be updated to take stock of these developments (both in terms of the actual calculation of methylation-based scores, as well as the information provided regarding applicability and limitations)?

*Response: The platform will be continually updated when robust DNAm-based predictors are generated by our groups and others. We have added a section called ‘Version Control’ within the ‘Implementation’ subsection in ‘Methods’. This section details who will be responsible for managing ‘MethylDetectR’, and that it will be updated every three months. We also invite other researchers to contact us if they wish to have their DNAm-based predictors added to ‘MethylDetectR’.*

**

***Version Control.** We will update ‘MethylDetectR’ every three months to include new DNAm-based predictors of human traits as they are generated by our own group and others. Updates will be managed by Robert F. Hillary or Riccardo E. Marioni. If researchers wish to have their predictors considered for inclusion in ‘MethylDetectR’, please use the corresponding author email address in this manuscript or the contact details available at the ‘MethylDetectR’ website ( https://www.ed.ac.uk/centre-genomic-medicine/research-groups/marioni-group/methyldetectr). The current and historical versions of ‘MethylDetectR’ are available in the Zenodo repository, updated versions will also be made available in this repository ( https://doi.org/10.5281/zenodo.4646300).*

*Further, we have created ‘Version Control’ action buttons at the start of each application, this details the history of updates to ‘MethylDetectR’ and indicates that its different versions can be accessed through our Zenodo repository. We also take this opportunity to highlight that our updated version of ‘MethylDetectR’ associated with this resubmission temporarily omits protein predictors from the dataset. The reason for this is that we are generating, and aim to publish, predictors for 109 blood protein levels (previously 27 proteins). We are committed to updating and managing ‘MethylDetectR’ as a live platform with new predictors added every three months. We will also continually monitor the platform to update information presented on the applications and website in light of feedback obtained from users and peers. The following text has been added the following text in Paragraph 1 of the ‘Discussion’:*

**

*“We will continue to update ‘MethylDetectR’ every three months with the aim of including new DNAm-based predictors of human traits when they come available”.*

**Comment 4: **The authors provide a link to their online interface for calculating DNAm scores. I would anticipate that, at least for research use, many studies would not be allowed to upload individual level DNAm data due to data protection policies, even when anonymized (some institutions have concerns that omics level data can never be truly anonymized), so it is useful that scripts are also provided to run the analyses locally.

*Response: We thank the reviewer for this comment. We also highlight that we have now included a data protection statement early within the text at the beginning of the ‘Methods’ section:*

**

*“ **Data Protection and Privacy**. No data are stored in ‘MethylDetectR’ and are deleted upon closing the applications. Applications are also timed out after three minutes of inactivity and are hosted on patched and secure servers within the Institute of Genetics and Cancer, University of Edinburgh. This research and translational tool complies with GDPR guidelines and has been designed to ensure the highest level of data security and privacy. ‘MethylDetectR’ and information on its usage are also available at the following website: https://www.ed.ac.uk/centre-genomic-medicine/research-groups/marioni-group/methyldetectr. Information relating to participant consent is also available at this website. Given that no data are stored, this information pertains to general risk surrounding the upload of biological data to online software and the measures taken to mitigate the risk of motivated intruders gaining access to such data.”*

**Comment 5:** Is it possible to upload potential covariates, such as cell-type, batch, ancestry etc.? How were these factors, especially cell-type heterogeneity, taken into account in the development of MethylDetectR DNAm scores?

*Response: ****When the predictors for lifestyle and biochemical traits were generated, they were pre-corrected for age, sex as well as genetic principal components to remove the potential influence of ancestry. We did not correct for cell-type heterogeneity in our training data. This is clarified with the addition of the following text under the ‘Lifestyle and biochemical traits’ subsection in ‘Methods’:*

**

*“In generating the predictors, phenotypes were pre-corrected to remove the influence of age, sex and ancestry using ten genetic principal components. Phenotypic data used to train the predictors were not corrected for cell-type heterogeneity.”*

**

*We also explored the addition of a ‘Covariates’ file to the ‘Calculate Your Scores’ application. When testing this, we felt that it may have been confusing for users given that not all users may wish to add covariates, and may wish to use some covariates to pre-correct DNAm data prior to the calculation of scores and also perhaps use other covariates to residualise the scores themselves. This is likely specific to each study. However, to address this excellent point raised by the reviewer, we have instead added text within the ‘Press Here for File Formats and Useful Links‘ ‘info’ button on the sidebar panel of the ‘Calculate Your Scores’ application to highlight that the user may wish to adjust the scores following their download for important covariates dependent on their study design or motivation to use ‘MethylDetectR’. We highlight cell-type heterogeneity as a key example of this discussion point:*

*In 'Calculate Your Scores': "Once you have downloaded the DNAm-based scores, you may wish to adjust them for covariates, such as cell type counts or proportions. Importantly, when the predictors were created, they were trained on phenotypic data that were not adjusted for cell-type heterogeneity. The need for covariate adjustments will be specific to the aims of each study.”*

**Comment 6:** Is this tool adequate for input datasets containing samples who may differ in characteristics from those upon which the algorithms were based (e.g. datasets of different ancestry)? Further, could the tool be utilized on child populations, where certain but not all scores would be applicable (e.g. BMI but not alcohol), if the algorithms were developed on adults? And regarding protein levels, how significant is the fact that these scores were developed from a sample of adults in old age? Can we assume that the same relationships between DNAm and protein levels hold across different life stages?

*Response: The reviewer raises excellent points of discussion. Our datasets were trained on an adult sample. It is possible that the predictors are not generalisable to different age groups and individuals with different ethnic backgrounds. The protein levels have been omitted temporarily from the platform but we will include a larger set of 109 proteins owing to refinements in the pipeline used to generate the protein predictors. In the future, we will adapt the platform to allow for comparisons of DNAm-based scores across samples. At the moment, this is challenging owing to variability in technical factors such as batch effects. We appreciate that we must acknowledge that the predictors may not be adequate for all age groups and datasets of different ancestry. To address these points, we have amended the text in Paragraph 3 of the ‘Discussion’ as follows:*

*“Distributions of DNAm values and subsequent DNAm-based scores may vary across different methylation datasets. In relation to the biochemical and lifestyle traits, the predictors were generated using an adult sample of individuals with European ancestry. Therefore, it is possible that the predictors may not be generalisable to datasets comprising different age ranges, such as cohorts of children, and individuals with different ancestries. Differences between datasets may result also arise from biological differences, for example cases for a given disease may have altered DNAm values for a number of probes relative to controls, or result from technical or normalisation differences. As a result, DNAm-based scores may vary greatly across datasets and projecting an individual onto a reference sample to view where their DNAm-based score would lie along the reference sample is therefore challenging. Future work will focus on developing methods which can appropriately account for variability across datasets and allow for a projection of individuals onto disparate DNAm samples or datasets.”*

**Comment 7: **It would help to add information in the demo about how to interpret the different modules (e.g. a walkthrough, or pop-up bubbles)

*Response: We agree with the reviewer that the demo version of the app should include detailed information on what each panel shows along with guidance on the interpretation of the plots. In addition to the other applications, we have now placed in ‘info’ action buttons on the sidebar of each panel to show information on what is being shown and how it may be interpreted in addition to key limitations.*

**Comment 8: **On a related note, is there a way to help users gauge how ‘reliable’ these different scores are? Some DNAm predictors (e.g. smoking) are more reliable and accurate than others, could this be indicated in the platform e.g. by visualizing this in the plots or adding text to aid interpretation (i.e. to what extent could a user interpret the percentiles for different scores with confidence)? I see in page 10 that error bars can be displayed, but (a) I do not see where the option to visualize these is in the online tool and this is also not explained in the manuscript; (b) I am assuming that these error bars are related to the input data, so a bit different from the point of indicating how good the scores themselves are. I assume that this can be partly gauged by the information on correlations in the fourth panel, although this includes only a selection of the DNAm scores.

*Response: We agree with the reviewer that information on the performance of the DNAm-based scores must be outlined. Panel 4 is designed to indicate how well DNAm-based scores correlate with phenotypic values of the traits in the large Generation Scotland sample. We have added appropriate text in ‘info’ buttons in the ‘MethylDetectR’ application and the demo version to remedy this issue and clarify the accuracy of the predictors in a large sample. Information on how well the predictors perform in the input dataset would require the upload of phenotypic data to the platform which is discouraged given data privacy concerns. The error bars in the plot are misleading in that they reflect interquartile ranges for the percentiles ranks attributed to cases for each predictor – this is clarified in the figure legend and in the ‘info’ button on the relevant panel. The figure legend of Figure 4B has been amended as follows:*

**

*“(B) The median percentile ranks for cases are plotted for selected traits of interest. The number shown in the circle reflects the median percentile, and the interquartile range of percentile ranks for cases is shown with the horizontal lines extending from the circle. Here, the median percentile ranks with respect to a number of physical traits are shown for cases of diabetes.”*

**

*Users of the ‘MethylDetectR’ and ‘MethylDetectR – Demo’ apps are also referred to Panel 4 within the information on the first panel in order to quickly indicate where they can go to view performance indicators of the predictors.*

**Comment 9: **Third panel how should users interpret differences in correlations between their input dataset and the reference GS dataset? What are the implications of these differences for interpreting their results (e.g. does it mean that scores should be interpreted with more caution or has no bearing on this)? For example, if the correlation between alcohol and smoking DNAm scores in the input dataset is 0.40, but in GS it is only 0.20, what does this mean for the user?

*Response: We thank the reviewer for highlighting this. As the reviewer indicates, the correlation structure can have important implications for interpreting how well the predictors perform across the input dataset and Generation Scotland participants. To resolve the concerns raised by the reviewer, we have added information in the application on Panel 3 accessible by clicking an ‘info’ button. This informs users that if the correlation structure differs greatly from that in Generation Scotland, then it may indicate that the general pattern of DNAm-based scores in their input dataset may differ with those in Generation Scotland. It may indicate that the characteristics of the two datasets differ from one another. Indeed, the performance of predictors in their own dataset may not be comparable to that of Generation Scotland.*

**Comment 10: **In the discussion, the authors state that ‘the MethylDetectR platform communicates key messages surrounding the development and present limitations of DNAm-based health profiling to the wider research community and public’ - where is this information provided? I cannot see it either in the Demo or Calculate your Scores platforms. This should be provided in the Demo, as the first ‘port of entry’ users should be using to get familiarized with the platform (and there should be a notice on the Calculate your Scores platform to first check out the Demo, for an example of correct use). This is a good place to walk users through the different functionalities/panels of the platform, and for each panel, to provide key information on interpretation (e.g. using case examples – “here is Joe, he is estimated to be xx years old and score within the xx percentile for yy variable. Based on this, we can conclude that xxx. Limitations to keep in mind are xxx. This information could be used for xxx applications”).

*Response: We agree with the reviewer and add information in the demo and main applications which can be accessed upon clicking an ‘info’ action button on each panel. This will allow the user, whatever their background, to immediately become familiarised with the calculation and the limitations of DNAm-based scores. Further, these action buttons provide a walkthrough of each panel. We thank the reviewer for their excellent and very helpful suggestions on how this information should be best portrayed.*

**Comment 11: **Similarly, in the conclusion, the authors state that “our platform provides an important translational tool which communicates state-of-the-art developments in relation to DNAm-based predictors of human traits and health… and highlights the applicability and limitations surrounding such predictors prior to their potential deployment in clinical assessment and management paradigms.” While the platform allows to calculate state-of-the-art scores, I cannot see anything related to communication of its uses and limitations.

*Response: As above, we have ensured to include this information within the applications themselves to resolve this shortcoming in the previous version of the platform.*

**Comment 12: **Generally, the manuscript refers to a lot of different files and links (e.g. the Demo, the actual platform, all the Zenodo materials). This relies on the idea that users in future will always refer to the manuscript and read it in detail before using the platform. I would suggest having an easy to find, centralized repository for all of these materials, or alternatively, to add the various links in the Demo/Calculate your Scores platforms (which should be linked somehow).

*Response: We agree with the reviewer that the various components of the platform should be easier to access. It was our aim to achieve this using the website. Necessarily, all files must be deposited to Zenodo, and example .csv files are not appropriate for the website given issues surrounding access i.e. text-to-speech conversion on our website. Based on the suggestion of the reviewer, we have included links to all elements of the platform, i.e. the other applications, paper, website and repository, within each application. This will make navigating the platform quick and easy for the user.*

**

**Comment 13: **The discussion is missing a section on the research and clinical applications of this platform. Can the authors provide concrete examples of where and how this information could be useful?

*Response: We thank the reviewer for highlighting this opportunity to improve communication of the potential utility of DNAm-based predictors. In addition to concerns raised by Reviewer 1, we acknowledge that, at present, the clinical applications of the DNAm-based scores are limited given that they cannot faithfully provide accurate estimates at an individual level. As above, we have added the following text in Paragraph 3 of the ‘Discussion’ section to reflect these concerns:*

**

*“Currently, the DNAm-based predictors in ‘MethylDetectR’ cannot make accurate predictions at an individual level and therefore cannot yet be reliably applied in a diagnostic or forensic context. Highly-accurate DNAm-based scores can aid in research environments as they may provide more accurate information than self-report data (6). For instance, the DNAm-based predictor of smoking can provide a more accurate profile of smoking history than responses from participants in questionnaires. Further, the use of DNAm-based scores for a variety of human traits can proxy many phenotypes such as biochemical and lifestyle traits using a single blood draw. Together, these data can help researchers determine relationships between putative risk factors and important health outcomes, and aid in patient stratification paradigms. Further, ‘MethylDetectR’ serves as an important translational tool showing an interactive, demo version of the platform and substantial information within each application regarding the interpretation and limitations of DNAm-based predictors. As these predictors become refined, they may be of clinical value. For instance, a blood-based DNAm test was recently developed that could detect five separate types of cancer up to four years before conventional diagnosis. The assay measured circulating tumour DNA methylation and predicted disease in 88% of post-diagnosis patients, with a specificity of 96%.”*

 Minor points

**Comment 14:** P.3: “In contrast to genetics, these molecular modifications are dynamic, tissue-specific and reversible” – would qualify this sentence a little bit. While it is correct that, overall, DNAm differ largely across tissues, some sites do show strong correspondence across multiple tissues. Similarly, it is unclear whether all DNAm changes are reversible (e.g. some smoking-related DNAm alterations persisting post-cessation).

*Response: We agree with the reviewer that this statement is not wholly accurate. As a result we have amended the text in Paragraph 1 of the ‘Introduction’ section as follows:*

*“The addition of these chemical tags can alter whether, and to what extent, a gene is active. In contrast to genetics, these molecular modifications are dynamic (3). Further, methylation at many CpG sites is tissue-specific though some show strong concordance across multiple tissues (4). In addition, CpG modifications induced through environmental factors, such as smoking, may be reversible or show persistent alterations (5).”*

**Comment 15: **P. 3: “Furthermore, there has been a rapid increase in the commercialisation and scalability of DNAm assays for direct-to-consumer use or for use in clinical, research or industrial settings.” Can the authors add references or include a couple of examples?

*Response: To remedy this, we have added the following reference and removed ‘rapid’ from this sentence:*

*https://www.nature.com/articles/s41576-020-0215-2*

**

*“Furthermore, there has been an increase in the commercialisation and scalability of DNAm assays for direct-to-consumer use or for use in clinical, research or industrial settings (new reference to https://www.nature.com/articles/s41576-020-0215-2).”*

**Comment 16: **P.3: “Blood DNAm data …provides a good index of the overall health status of the body.” Can the authors add a reference to support this?

*Response: We have amended the text to include the following reference in Paragraph 2 of the ‘Introduction’ section:*

**

*“Blood DNAm data is often used as it is minimally-invasive to collect and it provides a good index of the overall health status of the body (new reference to https://www.ncbi.nlm.nih.gov/pmc/articles/PMC7076122/)”.*

**Comment 17: **P. 4: “Lastly, the user can subset the reference samples by sex, age range or case vs. control status.” Case-control status on what variables?

*Response: Here, we refer to the case-control variables uploaded by the user. To make this clearer, we have amended the text as follows at the end of Paragraph 5 of the ‘Introduction’ section:*

*“Lastly, the user can subset the input sample by sex, age range or case vs. control status determined by the case-control variables uploaded by the user. Further, the user can subset the GS reference sample by age and sex.”*

**Comment 18: **P. 5: https://www.ed.ac.uk/centre-genomic-medicine/research-groups/marioni-group/methyldetectr the link provided has two attachments, which appear to contain the same information: MethylDetectR Participant Information Sheet and MethylDetectR Participant Consent Statement.

*Response: We sincerely thank the reviewer for spotting this error. The documents were accidentally duplicated on the website. This has been remedied to include the correct Participant Consent Statement.*

**Comment 19: **P. 5: in the explanation of methylation risk scores, the authors could point readers to a new review on the topic, including strengths and challenges of this approach (Huls & Czamara, 2020 Epigenetics)1.

*Response: We thank the reviewer for highlighting this review, we have included the following text under the section ‘Development of a DNAm-based predictor’ in ‘Methods’ to allow for its addition:*

**

*“Readers are referred to review on the development of DNAm-based scores and the challenges surrounding their generation (new reference to Huls & Czamara, 2020).”*

**Comment 20: **P. 6: Age predictor - can the authors specify the age range of the discovery data upon which the predictor was based?

*Response: We have added this information in the ‘Methods’ section under the ‘Age Predictor’ sub-section:*

*“The age predictor was generated using data from individuals with an age range of 2 to 104 years.”*

**Comment 21: **P. 6: Can the author provide a reference/specify how cutoffs were selected for their protein analyses (e.g. for training-test ratio, r and p thresholds etc.).

*Response: We have removed the blood protein predictors in the current version of ‘MethylDetectR’ but will include 109 blood protein predictors in the next update.*

# Peer review: Reviewer response for version 1

**DOI:** 10.21956/wellcomeopenres.18118.r42039

**Published:** 2021-01-22

**Authors:** Gemma Sharp

**Affiliations:** 1 MRC Integrative Epidemiology Unit, Population Health Sciences, University of Bristol, Bristol, UK

This paper describes a very useful and novel platform for generating DNA methylation-based predictors of traits from 450k/EPIC array data. The plots produced by the app are attractive and it is very pleasing to see that all code has been provided so that users can reproduce and adapt these outputs if they wish. I look forward to using the platform and thank and congratulate the authors on their great work.

 I have a few more specific comments and some ideas for how the paper and/or platform could possible be improved:

 The paper does a very good job of describing the potential for DNA methylation as an indicator or predictor of human traits and health. The motivation for doing this from a clinical point of view is well-argued.

 The need for the platform is clear. It saves the user a lot of time and effort in generating DNAm-based predictors of traits in their own data. While generating a DNAm-based predictor of epigenetic age has been possible for several years using the web-based platform developed by Horvath *et al*., this platform is unique in that it also includes six DNAm-based predictors of lifestyle and biochemical traits (alcohol consumption, body fat percentage, BMI, HDL cholesterol, smoking status and waist-to-hip ratio) and 27 predictors of blood protein levels related to inflammatory or neurological processes/diseases. The lifestyle predictors were generated in a large sample of individuals. The 27 protein predictors were generated in a much smaller sample, but replicated in an independent subset. 

 The platform consists of two applications, but it was a little unclear to me what these were. The first application is MethylDetectR - Calculate Your Scores. Is the second application "the main MethylDetectR application"?

 Users must upload their 450k or EPIC array data to the Calculate Your Scores application. The authors say that no data are stored by the application near the start of the paper. Later, in the Methods section, they provide more information on this and this section settled my concerns about data sharing of individual level data. Given that DNA methylation array data can potentially be used to identify individuals, data managers, legal teams and researchers may be understandably reluctant to upload data to an external website. Although it seems that this is probably a very low risk, it could be work highlighting this helpful section with a specific heading on data protection, and/or moving it to earlier in the paper.

 I agree that the utility of DNAm-based predictors lies in population-level research and are inaccurate and therefore of less use in predicting values at the individual level. If this platform can help illustrate that, it will be a useful teaching and learning aid. I suppose there's a concern that users may ignore that warning though and use the platform to make inferences about individuals. Are these caveats explained clearly within the app? It doesn't help that the app appears to provide predictions at the individual level, such as "we predict that you are 60 years old!" and I would strongly suggest changing this. Similarly in the discussion there is a paragraph that outlines the potential for these scores to be used in diagnosis/prognosis and forensics, which would require accurate prediction at the individual level. I suggest that this paragraph includes a caution that this is not possible using the scores generated by MethylDetectR. The issue with differences in distribution across datasets is explained well in the next paragraph, but I think the point (that MethylDetectR scores cannot be used to make predictions at the individual level) needs making explicitly.

 The upload limit is 3gb but files greater than 500mb "may take a considerable amount of time to upload". Is there any way to prepare files that could reduce this limitation? Does the website require data for every CpG on the array or just a subset that are useful in generating the scores? Should/could files be compressed?

 I was glad to see that an R script is provided for users who do not wish to/cannot upload DNAm data. The link provided goes to a repository of code and data. Would it be more appropriate/possible to release this as an R package?

 The demo version of the app is useful - please could it be mentioned nearer the start of the paper?

 The interface for the app itself would benefit from extra information being included on how files should be formatted, file size restrictions, etc. This would be more helpful for users than having to refer back to this paper or other instructions. Similarly, the plots generated in the MethylDetectR app are useful but some information on each tab explaining what this plot is showing is needed.

 Do the authors plan to update the app to include predictors for more traits as and when they are discovered?

 A small point about Figure 1 and Figure 2: I don't find that these figures add anything to the paper. For example, Figure 2 could be more clearly presented as a bulleted list, no extra information is conveyed by having the information in circles (and all information is already provided in the main text).

Are the conclusions about the tool and its performance adequately supported by the findings presented in the article?

Yes

Is the rationale for developing the new software tool clearly explained?

Yes

Is the description of the software tool technically sound?

Yes

Are sufficient details of the code, methods and analysis (if applicable) provided to allow replication of the software development and its use by others?

Yes

Is sufficient information provided to allow interpretation of the expected output datasets and any results generated using the tool?

Partly

Reviewer Expertise:

Epigenetic epidemiology, R, Shiny.

I confirm that I have read this submission and believe that I have an appropriate level of expertise to confirm that it is of an acceptable scientific standard, however I have significant reservations, as outlined above.

# Response

**Published:** 2021-03-30

**Authors:** Robert Hillary

**Affiliations:** University of Edinburgh, UK

**Comment 1:** The need for the platform is clear. It saves the user a lot of time and effort in generating DNAm-based predictors of traits in their own data. While generating a DNAm-based predictor of epigenetic age has been possible for several years using the web-based platform developed by Horvath *et al*., this platform is unique in that it also includes six DNAm-based predictors of lifestyle and biochemical traits (alcohol consumption, body fat percentage, BMI, HDL cholesterol, smoking status and waist-to-hip ratio) and 27 predictors of blood protein levels related to inflammatory or neurological processes/diseases. The lifestyle predictors were generated in a large sample of individuals. The 27 protein predictors were generated in a much smaller sample, but replicated in an independent subset. 

*Response: We thank the reviewer for their encouraging comments regarding this platform. We take this opportunity to highlight that our updated version of ‘MethylDetectR’ temporarily omits protein predictors from the dataset. The reason for this is that we are generating, and aim to publish, predictors for a larger set 109 blood protein levels including refinements in the pipeline used to generate the protein predictors. We are committed to updating and managing ‘MethylDetectR’. We will update ‘MethylDetectR’ every three months. We will include these 109 new predictors when they become available, likely in the next update. Owing to the temporary omission of blood protein predictors, references to these predictors in the text and figures have been removed. The manuscript has been updated accordingly throughout.*

**Comment 2:** The platform consists of two applications, but it was a little unclear to me what these were. The first application is MethylDetectR - Calculate Your Scores. Is the second application "the main MethylDetectR application"?

*Response: We thank the reviewer for highlighting this issue and agree that this must be clarified. The reviewer is correct, the first application is ‘MethylDetectR – Calculate Your Scores’. However, the use of this application is optional as users may wish to download the scripts to generate their own scores. Alternatively, the user may have to use the provided scripts if their DNAm file is too large for upload. Though, we now include an additional .csv file ‘Truncate_to_these_CpGs.csv’ to allow users to subset their DNAm file to those CpG sites used in the ‘MethylDetectR – Calculate Your Scores’ application. This all results in the main ‘MethylDetectR’ application being the ‘second’ application in the context of the entire platform and its pipeline. We have clarified this in the main text with the addition of the following text:*

*“* *Briefly, the ‘MethylDetectR’ platform consists of two applications. The first application named ‘MethylDetectR – Calculate Your Scores’ allows users to securely upload Illumina 450k or EPIC DNAm array data and obtain blood-based methylation predicted scores (or values) for the aforementioned traits. No data are stored by the application. Furthermore, predicted scores are often returned on arbitrary scales. The use of this application is optional as users may instead use R scripts which we have made publicly available if they so wish or if their DNAm files are too large for upload to the online application (>3 GB) (* *https://doi.org/10.5281/zenodo.4646300* *). However, users can access a file called ‘Truncate_to_these_CpGs.csv’ to subset the list of CpG sites in their DNAm files to those required by the ‘MethylDetectR – Calculate Your Scores’ application. This should reduce file size and upload time.*

*The second and main application named ‘MethylDetectR’ allows users to compare DNAm-derived scores for any individual in their input dataset against other individuals in the input dataset. Percentile ranks for individuals in the input dataset may be downloaded.”*

**Comment 3: **Users must upload their 450k or EPIC array data to the Calculate Your Scores application. The authors say that no data are stored by the application near the start of the paper. Later, in the Methods section, they provide more information on this and this section settled my concerns about data sharing of individual level data. Given that DNA methylation array data can potentially be used to identify individuals, data managers, legal teams and researchers may be understandably reluctant to upload data to an external website. Although it seems that this is probably a very low risk, it could be work highlighting this helpful section with a specific heading on data protection, and/or moving it to earlier in the paper.

*Response: We strongly agree with the reviewer that data protection and privacy concerns are of utmost importance in relation to this platform. We also agree that a dedicated section should be placed earlier in the manuscript discussing data protection and privacy aspects of the platform. As a result, we have added the following text at the top of the ‘Methods’ section giving data protection and privacy aspects their own dedicated section to appropriately reflect its importance:*

*“ **Data Protection and Privacy**. No data are stored in ‘MethylDetectR’ and are deleted upon closing the applications. Applications are also timed out after three minutes of inactivity and are hosted on patched and secure servers within the Institute of Genetics and Cancer, University of Edinburgh. This research and translational tool complies with GDPR guidelines and has been designed to ensure the highest level of data security and privacy. ‘MethylDetectR’ and information on its usage are also available at the following website:* *https://www.ed.ac.uk/centre-genomic-medicine/research-groups/marioni-group/methyldetectr* *. Information relating to participant consent is also available at this website. Given that no data are stored, this information pertains to general risk surrounding the upload of biological data to online software and the measures taken to mitigate the risk of motivated intruders gaining access to such data.”*

**Comment 4:** I agree that the utility of DNAm-based predictors lies in population-level research and are inaccurate and therefore of less use in predicting values at the individual level. If this platform can help illustrate that, it will be a useful teaching and learning aid. I suppose there's a concern that users may ignore that warning though and use the platform to make inferences about individuals. Are these caveats explained clearly within the app? It doesn't help that the app appears to provide predictions at the individual level, such as "we predict that you are 60 years old!" and I would strongly suggest changing this. Similarly, in the discussion, there is a paragraph that outlines the potential for these scores to be used in diagnosis/prognosis and forensics, which would require accurate prediction at the individual level. I suggest that this paragraph includes a caution that this is not possible using the scores generated by MethylDetectR. The issue with differences in distribution across datasets is explained well in the next paragraph, but I think the point (that MethylDetectR scores cannot be used to make predictions at the individual level) needs making explicitly.

Response: We agree with the reviewer that the platform provides an opportunity to communicate the message that DNAm-based predictors work well at a population level, but cannot make accurate predictions at an individual level. This has major implications for their use in clinical settings. We, therefore, take the following actions. First, we change the text in the first panel of ‘MethylDetectR’ and ‘MethylDetectR – Demo’ to include the following message: “Epigenetic Clock (Zhang) age estimate: X years*” and include underneath “*All methylation-based predictors in ‘MethylDetectR’ can make inaccurate predictions at an individual level, limiting clinical utility. However, they can work well at the population level and will improve further with more refined prediction methods and larger-scale studies”. This ensures that the message is communicated explicitly at the first instance of the application.

**

*Second, we amend text in Paragraph 2 of the ‘Discussion’ section to mention that DNAm-based scores in ‘MethylDetectR’ cannot be used in diagnosis/prognosis and forensics:*

**

*“Currently, the DNAm-based predictors in ‘MethylDetectR’ cannot make consistently accurate predictions at an individual level and therefore cannot be reliably applied in a diagnostic or forensic context.”*

*Third, we remove the following text from the same paragraph:*

**

*“DNAm-based predictors of human traits, such as chronological age and body mass index, may also aid in forensic contexts.”*

**Comment 5: **The upload limit is 3gb but files greater than 500mb "may take a considerable amount of time to upload". Is there any way to prepare files that could reduce this limitation? Does the website require data for every CpG on the array or just a subset that are useful in generating the scores? Should/could files be compressed?

*Response: We recognise this as a current limitation of the software. To resolve this issue, we provide a list of CpG sites (‘Truncate_to_these_CpGs.csv’) which the user can use to truncate their DNAm dataset prior to upload. This is included in the Zenodo repository and also clearly detailed in the ‘Press Here to Format Files and Useful Links’ action button in the ‘Calculate Your Scores’ application.*

**Comment 6: **I was glad to see that an R script is provided for users who do not wish to/cannot upload DNAm data. The link provided goes to a repository of code and data. Would it be more appropriate/possible to release this as an R package?

*Response: We thank the reviewer for this excellent suggestion. At the moment, we feel that the provided code and applications achieve a similar goal as an R package.  Nonetheless, in the future, we aim to refine the platform by developing methods that can allow for comparisons of DNAm-based scores across different cohorts. Currently, this is challenging owing to variability in technical factors across different DNAm datasets, such as batch effects. As a result, the application allows only for the calculation of DNAm-based scores within the input dataset. Furthermore, we hope to release an R package that can allow for the projection of DNAm-based scores onto other cohorts, such as those in Generation Scotland and allow for visual comparisons between distributions of scores in the input cohort and those within Generation Scotland.*

**Comment 7:** The demo version of the app is useful - please could it be mentioned nearer the start of the paper?

*Response: The demo version of the app is now mentioned at the start of the paper in Paragraph 4 of the ‘Introduction’ section. It was previously mentioned in the ‘Methods’ section.*

**Comment 8:** The interface for the app itself would benefit from extra information being included on how files should be formatted, file size restrictions, etc. This would be more helpful for users than having to refer back to this paper or other instructions. Similarly, the plots generated in the MethylDetectR app are useful but some information on each tab explaining what this plot is showing is needed.

*Response: We thank the reviewer for suggesting this useful addition to the platform. We have now included ‘info’ action buttons on each panel of ‘MethylDetectR’, ‘MethylDetectR – Demo’ and on ‘MethylDetectR – Calculate Your Scores’. These action buttons provide information on what the panels show and how to format files. We also include links to the main website and links to the other apps so navigation between the different components of the platform is simplified.*

**Comment 9**: Do the authors plan to update the app to include predictors for more traits as and when they are discovered?

*Response: Yes, we will continue to update the applications and platform to include a larger set of DNAm-based predictors of protein levels. We will add in predictors of 109 blood proteins and include robust DNAm-based predictors of human traits generated in our group and others. We will update ‘MethylDetectR’ every three months. To clarify this, we have added a ‘Version Control’ paragraph to the ‘Implementation’ subsection of ‘Methods’:*

**

*“ **Version Control.** We will update ‘MethylDetectR’ every three months to include new DNAm-based predictors of human traits as they are generated by our own group and others. Updates will be managed by Robert F. Hillary or Riccardo E. Marioni. If researchers wish to have their predictors considered for inclusion in ‘MethylDetectR’, please use the corresponding author email address in this manuscript or the contact details available at the ‘MethylDetectR’ website ( https://www.ed.ac.uk/centre-genomic-medicine/research-groups/marioni-group/methyldetectr). The current and historical versions of ‘MethylDetectR’ will be available in the Zenodo repository, updated versions will also be made available in this repository ( https://doi.org/10.5281/zenodo.4646300)”.*

*We have also added the following text to Paragraph 1 of the ‘Discussion’:*

*“We will continue to update ‘MethylDetectR’ every three months with the aim of including new DNAm-based predictors of human traits when they come available”.*

**Comment 10**: A small point about Figure 1 and Figure 2: I don't find that these figures add anything to the paper. For example, Figure 2 could be more clearly presented as a bulleted list, no extra information is conveyed by having the information in circles (and all information is already provided in the main text).

*Response: We agree with the reviewer that these figures are not essential for the paper or the platform. As a result, we have removed them and updated figure annotations accordingly.*
